# Brain Resident Ly6C^hi^ Monocytes Are Necessary for Maintaining Adult Hippocampal Neurogenesis

**DOI:** 10.14336/AD.2024.0835

**Published:** 2025-11-18

**Authors:** Yiran Huang, Nan Gao, Boren Liu, Weili Luo, Jianfei Chen, Yan Chen, Yong Bi, Zikai Zhou

**Affiliations:** ^1^School of Pharmacy, Guizhou Medical University, Guizhou, China.; ^2^Zhongshan Institute for Drug Discovery, Shanghai Institute of Materia Medica, Chinese Academy of Sciences, Zhongshan, China.; ^3^School of Pharmaceutical Sciences, Southern Medical University, Guangzhou, China.; ^4^University of Chinese Academy of Sciences, Beijing, China.; ^5^Shanghai University of Medicine and Health Sciences Affiliated Zhoupu Hospital, Shanghai, China.; ^6^State Key Laboratory of Drug Research, Shanghai Institute of Materia Medica, Chinese Academy of Sciences, Shanghai, China.

**Keywords:** Monocytes, Stress, Bone marrow homing, Adult hippocampal neurogenesis

## Abstract

Adult hippocampal neurogenesis (AHN) is crucial to various brain functions. Neurodegeneration, neuroinflammation and stress can impair AHN, contributing to the development of neurological and psychiatric disorders. Stress is known to extensively affect both the brain and peripheral immune system. However, the cellular and molecular mechanisms underlying stress-induced impairments in AHN remain unclear. In this study, we found that, unlike neuroinflammatory conditions, stress significantly inhibited AHN independently of microglial activation, suggesting a novel mechanism mediating stress-impaired AHN. Since stress modulates peripheral immune cells, we examined the distribution of immune cells infiltrating the brain. We found a significant decrease of infiltrated Ly6C^hi^ monocytes in the brain parenchyma. In the blood, adoptively transferred ZsGreen^+^ Ly6C^hi^ monocytes drastically reduced due to stress-induced homing to the bone marrow. Adrenalectomy (ADX) experiments revealed that monocyte homing is regulated by glucocorticoid and may cause impairments in AHN. Depleting peripheral circulating monocytes reduced brain-resident Ly6C^hi^ monocytes and replicated the stress-induced inhibition of AHN, independent of microglia activation. RNA sequencing analysis of Ly6C^hi^ monocytes revealed a stress-induced transcriptional profile, suggesting their supportive role in neuronal functions. Together, these findings demonstrate a novel and essential role of brain resident Ly6C^hi^ monocytes in maintaining AHN at basal level, which is important for brain functions.

## INTRODUCTION

Adult hippocampal neurogenesis (AHN) is crucial for various higher brain functions and mental health, particularly in aging [1]. Newly generated neurons integrate into existing neural circuits, facilitating memory encoding and retrieval [2]. Chronic stress elevates cortisol levels, the primary stress hormone, which extensively impairs brain and immune functions. This stress-induced maladaptive neuroplasticity accelerates cognitive decline and increases susceptibility to diseases such as anxiety, depression, and post-traumatic stress disorder (PTSD) [3]. Extensive experimental evidence underscores AHN's role in promoting stress resilience, reducing vulnerability to stress-related psychiatric disorders such as depression and anxiety, and diminishing the risk of Alzheimer's disease (AD) and dementia [4]. Neuropharmacological research further corroborates these findings, indicating that enhanced AHN is associated with antidepressant effects, while reduced neurogenesis correlates with increased vulnerability to stress-induced mood disturbances. Collectively, these findings highlight the importance of AHN in maintaining cognitive function and resilience against stress-related disorders.

In the aging population, deficits in AHN are believed to partially contribute to the comorbidity of major depressive disorder (MDD) and AD or dementia [5]. Multiple longitudinal studies and meta-analyses have found that individuals with a history of depression have approximately twice the risk of developing dementia compared to those without depression [6, 7]. This association persists even when depression occurs 25 years or more before the onset of dementia symptoms [8]. Moreover, the risk of dementia increases with the severity and duration of depressive symptoms [7]. A meta-analysis estimated that 8.6% of all new dementia cases and 10.8% of new AD cases could be attributed to depression [6]. Currently, potential mechanisms linking depression and dementia are thought to include hippocampal atrophy, increased neuroinflammation, oxidative stress, and shared genetic vulnerabilities [9].

Emerging evidence suggests that stress-induced impairment of AHN may serve as a mechanistic link between MDD and AD. However, the understanding of AHN alone cannot fully explain this correlation. For instance, although cortisol is known to impair immune function and reduce neuroinflammation, AD is characterized by significant intracerebral inflammation [10]. Therefore, further investigation is crucial to comprehensively elucidate the mechanisms underlying stress-induced AHN impairment and its role in neuropsychiatric disorders.

Recent studies have uncovered direct modulatory roles of peripheral immune cells on neurons and glial cells in the brain under pathological conditions [11]. Given the extensive impact of stress on peripheral immunity, exploring the involvement of immune cells in AHN is crucial. Monocytes, a key subset of innate immune cells, are essential for immune surveillance and defense against infections [12]. While their role in the progression of neuropsychiatric diseases has been reported, it remains unclear. This study investigates how stress affects monocyte behavior, particularly their homing to the bone marrow and its subsequent impact on AHN. Understanding these processes may lead to novel therapeutic strategies for stress-related disorders by leveraging monocytes' potential in maintaining neurogenesis.

## MATERIALS AND METHODS

### Animals

C57BL/6, APP/PS1, B6-G/R, Lyz2-cre, and Rosa26-LSL-tdTomato were purchased from The Jackson Laboratory. Genotyping for each strain was performed following the protocols recommended by The Jackson Laboratory website. Relevant mice were crossed to generate Lyz2-tdtomato and respective littermate controls. All experiments were performed on female 8- to 16-week-old mice with age- and sex-matched controls. All mice were housed on a 12h/12h light/dark cycle at 22°C with unrestricted access to food and water. Where appropriate, mice were randomly assigned to groups, and experiments were performed in a blind manner. All the animal experimental procedures complied with the institutional ethical guidelines and were approved by the Institutional Animal Care and Use Committee (IACUC) of Zhongshan Institute for Drug Discovery.

**Table 1:** Reagents.

REAGENT or RESOURCE	SOURCE	IDENTIFIER
**Rabbit anti-mouse DCX**	CST	4604S
**Rabbit anti-mouse Iba1**	proteintech	10904-1-AP
**Rat anti-mouse CD31**	abcam	AB256569
**Rabbit anti-mouse S100A9**	CST	73425S
**Anti-rabbit IgG**	Abbkine	A23310
**Anti-rat IgG**	Abbkine	A23240
**FITC anti-mouse NK-1.1**	Biolegend	108706
**PE/Cyanine5 anti-mouse F4/80**	Biolegend	123111
**PerCP/Cyanine5.5 anti-mouse Ly-6C**	Biolegend	128012
**Brilliant Violet 510™ anti-mouse I-A/I-E**	Biolegend	107635
**PerCP anti-mouse I-A/I-E**	Biolegend	107624
**Brilliant Violet 605™ anti-mouse CX3CR1**	Biolegend	149027
**PE/Cyanine7 anti-mouse/human CD11b**	Biolegend	101216
**Brilliant Violet 421™ anti-mouse/human CD11b**	Biolegend	101235
**APC anti-mouse Ly-6G**	Biolegend	127614
**PE anti-mouse MERTK**	Biolegend	151506
**APC/Cyanine7 anti-mouse CD38**	Biolegend	102727
**Brilliant Violet 421™ anti-mouse CD115**	Biolegend	135513
**FITC anti-mouse CD19**	BD	557398
**FITC anti-mouse CD3**	BD	561798
**FITC anti-mouse CD90.2**	BD	553003
**APC anti-mouse TCR beta**	Tonbo	20-5961-U100
**PE anti-mouse CD115**	Tonbo	50-1152-U100
**PE anti-mouse S100A9**	CST	93941S
**APC anti-mouse CD206**	Biolegend	141708
**APC/Cyanine7 anti-mouse CD45**	BD	557659
**FITC anti-mouse CD45**	biogems	07512-50-100
**Anti-mouse CD16/32**	Biolegend	156603
**7-AAD Viability Staining Solution**	Thermo Fisher	00-6993-50
**Fixable Viability Stain 700**	BD	564997
**PBS**	Gibco	C10010500BT
**HBSS**	YEASEN	60149ES76
**PBS (10X)**	Sangon	E607016-0500
**Cell Staining Buffer**	Biolegend	420201
**RBC Lysis Buffer (10X)**	Biolegend	420302
**Percoll**	Cytiva	17089102
**RPMI 1640**	Gibco	C22400500BT
**Dextrose Citrate Solution B (ACD, sterile)**	Beyotime	ST496
**Triton™ X-100**	Aladdin	T109026
**BSA**	Biofroxx	4240GR005-3
**FBS**	Meilunbio	PWL001-3
**Collagenase Type IV**	Sigma	V900893
**Collagenase Type I**	Sigma	V900891
**Deoxyribonuclease I**	Sigma	DN25
**Tissue-Tek O.C.T. Compound**	SAKURA	4583
**Clodronate Liposomes**	YEASEN	40337ES08

### Learned helplessness

Learned helplessness (LH) was induced using a standard inescapable foot shock protocol, as described previously [13]. Briefly, 8-10-week-old female wild-type C57BL/6 mice were randomly divided into non-stressed (NS) and foot-shock (FS) groups. Mice were housed adaptively for 7 days prior to the procedure, with 5 mice per cage. For LH induction, FS mice were placed in a two-compartment shuttle box with the passage between chambers closed. After a 1 min adaptation period, mice received 60 min of random, inescapable foot shocks (0.3 mA, 1-3 s duration, 1-15 s randomized intervals). NS mice were placed in the box for 61 min without shocks. The NS group was subjected to the same non-target stressors as the FS group, with shocks omitted. This procedure was repeated for 2 consecutive days. 24 hours after the last inescapable foot shocks, FS mice underwent LH testing in the shuttle box with the passage open. After 1 min adaptation, mice received 30 trials of escapable foot shocks (0.3 mA, 10 s duration, 30 s intervals). A cue light signaled 5 s before each shock. Avoidance was recorded if mice shuttled to the other compartment within the 5 s cue. Escape latency was measured if mice shuttled during the 10 s shock. Failure to shuttle within 10 s was counted as an escape failure. Shocks were immediately terminated upon shuttling. Based on the testing data, mice were classified as LH or non-learned helplessness (NLH) using predetermined criteria of escape failure number, escape latency, and linear discriminant equations [14]. Mice with LH > R were defined as LH, while mice with R > LH were defined as NLH, where R = -2.28185 + (2.17825 * escape latency) + (-0.57025 * escape failures) and LH = -26.97824 + (-0.10226 * escape latency) + (2.15966 * escape failures).

### Chronic restraint stress

Chronic restraint stress (CRS) was induced using a standard protocol. Briefly, 8-10-week-old female wild-type C57BL/6 mice were randomly divided into NS and CRS groups. Mice were housed in groups of five per cage and allowed to acclimate for 7 days before the procedure, with 5 mice per cage. Restraint tubes were prepared by drilling 0.5 cm diameter breathing holes (0.5 cm diameter) in the wall and lid of 50 mL conical tubes, spaced 2 cm apart. Before each restraint session, tubes were sterilized with 75% ethanol and exposed to UV irradiation for 15 min. For CRS induction, mice were placed in the restraint tubes for 2 hours per day (12:00-14:00) for 21 consecutive days. The procedure was consistently performed under the same conditions. NS mice were not restrained but were deprived of food and water for 2 hours (12:00 -14:00) each day. The NS group was subjected to the same non-target stressors as the CRS group.

### Adrenalectomy

Eight- to ten-week-old female wild-type C57BL/6 mice were randomly assigned to either sham surgery or adrenalectomy (ADX) groups. Mice were housed 6 per cage and acclimated for 7 days prior to surgery. ADX mice were anesthetized with 2.5% isoflurane delivered in 0.5 L/min of oxygen via a nose cone. Erythromycin ophthalmic ointment was applied to the eyes to prevent corneal drying. Bilateral adrenalectomy was performed through dorsolateral subcostal incisions. Hair was removed from the surgical site, which was then disinfected with 75% ethanol. Skin and muscle were incised to expose the kidneys and adrenal glands. The adrenal glands were carefully removed with fine forceps. The muscle and skin were then closed with absorbable sutures. Sham surgeries were performed using the same procedure, but without adrenal gland removal. After surgery, both groups received saline to aid recovery. Mice were allowed to recover for at least 14 days under standard housing and ad libitum access to food and water before further experiments.

### Lipopolysaccharide-induced neuroinflammation

8- to 10-week-old female wild-type C57BL/6 mice were randomly assigned to receive either intracerebro-ventricular injection (i.c.v.) of lipopolysaccharide (LPS) or phosphate-buffered saline (PBS) as a control. Mice were housed six per cage and allowed to acclimate for 7 days prior to surgery. During the inactive phase, LPS i.c.v. mice were anesthetized with 2.5% isoflurane delivered in 0.5 L/min oxygen and placed in a stereotaxic frame. Erythromycin ophthalmic ointment was applied to the eyes to prevent corneal drying. The surgical site was shaved and disinfected with 75% ethanol. A midline incision was made to expose the skull, which was kept moist with sterile saline. Bregma and lambda were identified, and the skull was adjusted to ensure they were in the same horizontal plane, with points 1.3 mm lateral to bregma also leveled. Using bregma as the reference point, coordinates (ML: 1.0 mm, AP: -0.5 mm, DV: -2.3 mm) were used to mark the injection site 0.5 mm posterior and 1.0 mm right of bregma. A small hole was carefully drilled through the skull at the marked location without damaging the underlying brain tissue. A glass micropipette was used to inject 5 μL of LPS (0.5 mg/mL) or sterile PBS at a rate of 1 μL/min. The micropipette was left in place for 2 min, then raised to DV: -1.15 mm and left for another minute before withdrawing. The incision was closed with absorbable sutures and disinfected with iodine. After surgery, mice were placed on a heating pad and monitored until fully recovered and ambulatory.

### Adoptive transfer of immune cells

For isolation of ZsGreen expressing splenocytes and bone marrow cells, female B6-G/R mice aged 8-10 weeks were euthanized by cervical dislocation under anesthesia. Spleens, femurs, and tibias were surgically removed. After lysing red blood cells, cell counting, and viability assessments were performed. ZsGreen expressions were assessed in both splenocytes and bone marrow cells. Suspensions were adjusted to 1×10^8^ cells/mL in cold PBS and combined in a 1:1 ratio to generate a single-cell suspension of splenocytes and bone marrow cells. Wild-type female C57BL/6 mice aged 8-10 weeks received 1×10^7^ ZsGreen^+^ immune cells via tail vein injection in 100 μL PBS. Recipient mice were allowed to recover for at least 28 days under standard housing conditions before further experiments.

### Depletion of Monocytes

Clodronate liposomes (CLO, 10 mL/kg) were administered intravenously via the lateral tail vein every 7 days for a period of 21 days to deplete circulating monocytes. Control mice received PBS at the same time points. The efficiency of CLO in depleting monocyte in normal mice was evaluated by flow cytometry analysis of peripheral blood.

### Immunofluorescence

Mouse brains were surgically removed and fixed in 4% paraformaldehyde at 4°C for 48 hours. After fixation, brains were dehydrated in 30% sucrose solution for 48 h, then transferred to fresh 30% sucrose until they sank to the bottom of the tube. Dehydrated brains were embedded in O.C.T. compound, taking care to remove any bubbles around the tissue, and immediately frozen at -80°C. Coronal sections of 25 or 40 μm thickness were cut using a cryostat and mounted on adhesive slides. Slides were air-dried at room temperature, protected from light. For washing, slides were placed in a staining dish filled with PBS and gently agitated on a shaker at room temperature for three 5-minute intervals. Sections were permeabilized by shaking in PBS with 0.5% Triton X-100 for 30 minutes at room temperature. After removing excess liquid around the sections, a hydrophobic barrier pen was used to encircle the tissue. Blocking was performed by adding 200 μL of PBS with 0.5% Triton X-100, 5% FBS, and 5% BSA to each encircled area. Samples were incubated for 2 hours at room temperature in a humidified chamber. Primary antibodies (DCX, Iba1, CD31, S100A9, etc.) were diluted 1:500 in PBS with 0.1% Triton X-100 and 5% BSA. After removing the blocking solution, 200 μL of primary antibody solution was added to each section and incubated overnight at 4°C in a humidified chamber. Secondary antibody control sections received an equal volume of PBS with 0.1% Triton X-100 and 5% BSA without primary antibody. The next day, primary antibody solution was removed, and slides were washed three times for 5 minutes each in PBS with 0.1% Triton X-100. Fluorescent secondary antibodies were diluted 1:500 in PBS with 0.1% Triton X-100 and 5% BSA. After re-encircling the sections, 200 μL of secondary antibody solution was added and incubated for 2 h at room temperature. Following secondary antibody incubation, slides were washed 3 times for 5 min each in PBS, protected from light. Excess liquid was removed, and slides were air-dried before mounting with DAPI-containing mounting medium and coverslips. Slides were imaged using a confocal microscope.

### Imaging

For low magnification images of the hippocampal, images were taken by slide scanner (Olympus SLIDEVIEW VS200). For high magnification images of the neuron or microglia, all confocal images containing regions of interests were taken by confocal microscopy (Olympus FLUOVIEW3000) under a × 20 objective, 2 × zoom in Z-resolution 0.5 mm/slice and then loaded the maximum intensity projection. All files were saved in Tiff format and post-processed using ImageJ (FIJI). Image analyses were performed blind to the experimental group.

### Cell counting

Immunofluorescence staining was performed on sections to detect the DCX^+^ cells, Iba1^+^ cells, S100A9^+^ cells and Lyz2^+^ cells. Cell counting in DG was performed on 5 sections per mouse, spanning from anterior to posterior. The number of cells in each DG section was divided by the DG volume, calculated as the area of the granule cell layer (GCL) measured by DAPI staining, divided by the section thickness, to obtain the cell density. The average cell density across the 5 sections was then calculated for each animal.

### Morphology analysis

DCX^+^ cells, which exhibit a dendritic structure, were captured and analyzed using the AnalyzeSkeleton plugin in ImageJ to trace neuron cell bodies and dendrites. Similarly, microglial morphology was quantified using the AnalyzeSkeleton and FracLac plugins in ImageJ. Briefly, fluorescent photomicrographs were converted to grayscale images and binarized by thresholding. Binarized neurons or microglia were then skeletonized, and the number of branches and total branch length were measured for each individual cell. A total of 15 cells from 3 slices were counted per animal.

### Flow cytometry

To isolate splenocytes, spleens were gently ground through a 70 μm cell strainer placed on a 50 mL centrifuge tube, using a 1 mL syringe plunger. The strainer was pre-wetted with 1 mL ice-cold RPMI 1640 medium. Additional cold medium was added during grinding to minimize mechanical damage to cells. For bone marrow cells, the ends of femurs were cut, and the marrow cavity was flushed with cold RPMI 1640 using a syringe. The marrow was dissociated by repeated aspiration.

To isolate brain immune cells, brains were bisected along the midline and the olfactory bulbs, cerebellum, and brainstem were removed. Each hemisphere was minced into 1-2 mm pieces using scissors and placed in a 15 mL tube with 1 mL pre-warmed RPMI medium and 0.5 mL enzyme mix (Dilute 90 U mL^-1^ DNase I, 30 U mL^-1^ collagenase type I and 1200 U mL^-1^ collagenase type IV in 1× HBSS). Tissue was digested for 30 min at 37°C with trituration every 5 min. Digestion was stopped by adding 7 mL cold RPMI, followed by 3 mL SIP solution to create a 30% SIP mixture. This was carefully layered over 2 mL 70% SIP in a 15 mL tube. After centrifuging at 500 g for 30 min at 18°C with no brake, the myelin layer and interphase cells were collected, filtered through a 40 μm strainer, and washed with 10 mL cold staining buffer.

Red blood cells were lysed using 1× lysis buffer (diluted from 10× stock) at a ratio of 2 mL per 100 μL blood, with incubation for 5 min at room temperature. After centrifuging at 350×g for 5 min at 4°C, the lysis step was repeated, and cells were resuspended in 5 mL cold staining buffer and filtered. Splenocytes and bone marrow cells were similarly lysed using 5 mL 1× buffer per cell pellet for 5 min on ice, then washed with 30 mL cold PBS.

For staining, cell suspensions were adjusted to 4×10^7^ cells/mL in cold staining buffer. Fc receptors were blocked with 0.5 μL blocking reagent per 50 μL cell suspension for 15 min at 4°C. Antibodies were diluted 1:100 in cold staining buffer. 50 μL diluted antibody was added per sample and incubated for 40 min at 4°C, protected from light. Cells were washed with 1 mL cold staining buffer and centrifuged at 350×g for 5 min at 4°C. Pellets were resuspended in 500 μL cold staining buffer for flow cytometry.

Cells were identified as (1) Microglia (CD45^int^ CD11b^+^), (2) T cells (CD45^hi^ CD11b^-^ Lineage^+^ MHC-II^-^), (3) B cells (CD45^hi^ CD11b^-^ Lineage^+^ MHC-II^+^), (4) neutrophils (CD45^hi^ CD11b^+^ Lineage^-^ CX3CR1^-^ Ly6G^+^), (5) Ly6C^hi^ monocytes (CD45^hi^ CD11b^+^ Lineage^-^ Ly6G^-^ CX3CR1^+^ Ly6C^hi^), (6) Lineage was defined as: CD3, CD90.2, CD19, NK1.1.

**Figure 1.**
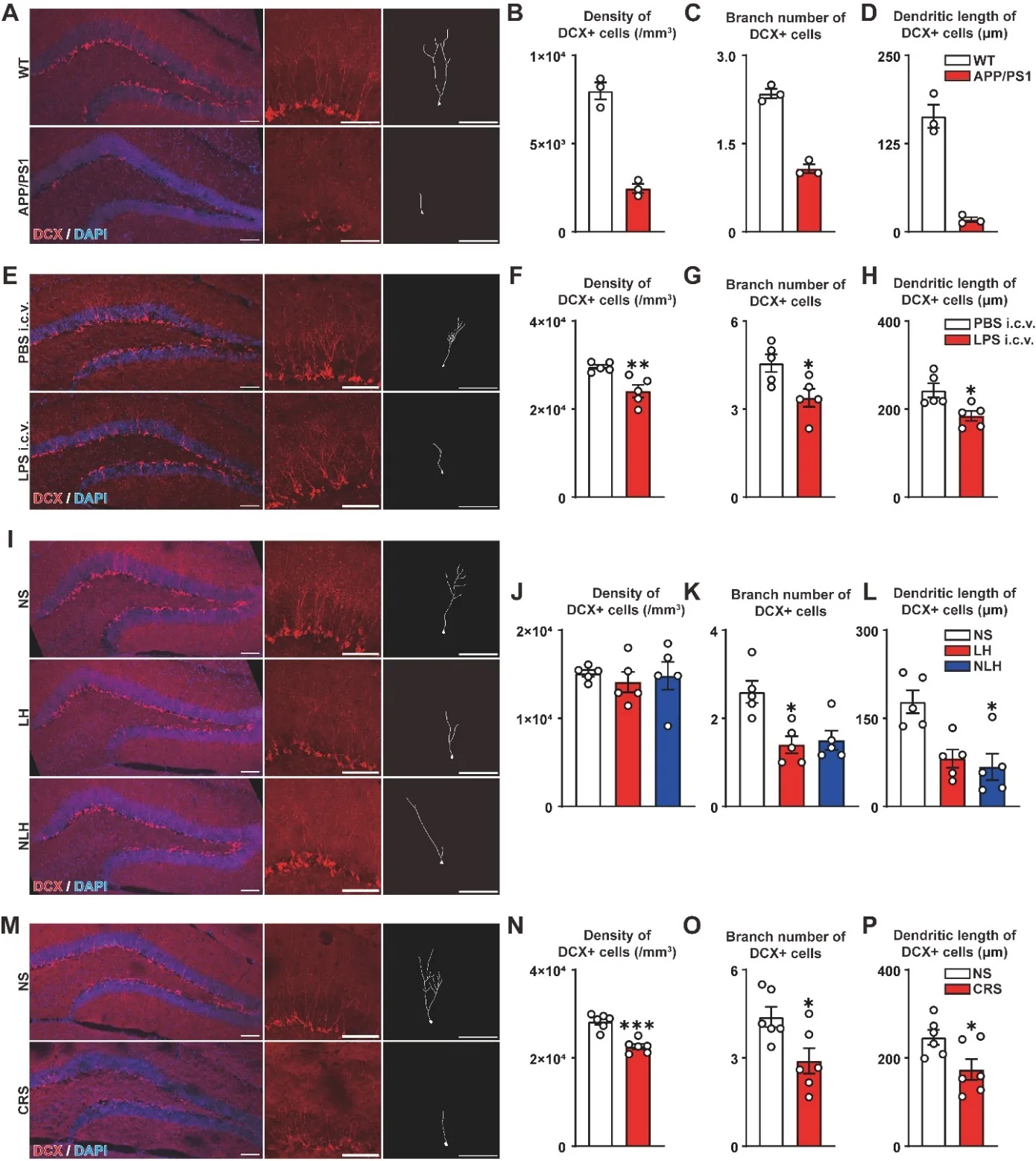
**Neurodegeneration, neuroinflammation and stress inhibit adult hippocampal neurogenesis**. (**A**) Hippocampal coronal sections of the brains of mice in the WT and APP/PS1 groups were stained with DCX antibody. Scale bar = 100 μm, Scale bar of morphological analysis = 100 μm. (B, C, D) Quantification of the density of DCX^+^ newborn immature neuron (B), average dendritic branch number (C), and average dendritic branch length (D). n = 3 mice in each group. (**E**) Hippocampal coronal sections of the brains of mice in the PBS i.c.v. and LPS i.c.v. groups were stained with DCX antibody. Scale bar = 100 μm, Scale bar of morphological analysis = 100 μm. (F, G, H) Quantification of the density of DCX^+^ newborn immature neuron (F), average dendritic branch number (G), and average dendritic branch length (H). n = 5 mice in each group. (**I**) Hippocampal coronal sections of the brains of mice in the NS, LH and NLH groups were stained with DCX antibody. Scale bar = 100 μm, Scale bar of morphological analysis = 100 μm. (J, K, L) Quantification of the density of DCX^+^ newborn immature neuron (J), average dendritic branch number (K), and average dendritic branch length (L). n = 5 mice in each group. (**M**) Hippocampal coronal sections of the brains of mice in the NS and CRS groups were stained with DCX antibody. Scale bar = 100 μm, Scale bar of morphological analysis = 100 μm. (N, O, P) Quantification of the density of DCX^+^ newborn immature neuron (N), average dendritic branch number (O), and average dendritic branch length (P). n = 6 mice in each group. For (B-D, F-H, J-L, N-P), data are represented as mean ± SEM. Statistical analysis was performed using Kruskal-Wallis test (J-L), Mann Whitney test (B-D, F-H) and two-tailed unpaired t-tests (N-P); *p < 0.05, **p < 0.01, ***p < 0.001.

### Cell sorting and Bulk RNA-seq

After isolating blood cells as described above, cell suspensions were stained to identify the Ly6C^hi^ monocyte populations (each sample was pooled from n=5 mice). Cells were sorted on a BECKMAN cell sorter directly into Trizol, flash-frozen and stored at -80°C. Total RNA was isolated using the RNeasy Mini Kit (Qiagen). RNA quality was assessed by gel electrophoresis and Qubit (Thermo). Strand-specific RNA-seq libraries were constructed using the TruSeq RNA sample preparation kit (Illumina) and sequenced on an Illumina Novaseq 6000 instrument. The raw data was processed using Skewer and data quality was assessed with FastQC v0.11.2. Transcript expression levels were quantified as Fragments Per Kilobase of exon model per Million mapped reads (FPKM) using a Perl script. Differential expression transcripts (DETs) were determined using the MA-plot-based method with Random Sampling (MARS) model in the DEGseq package. DETs were defined as those with a value < 0.05, an absolute fold change > 1.5 and detection in at least two samples. Gene Ontology (GO) and Kyoto Encyclopedia of Genes and Genomes (KEGG) pathway enrichment analyses were performed on the identified DETs to elucidate their biological functions and signaling pathways.

### Statistical analysis

Data are presented as mean ± standard error of the mean (SEM). Comparisons between two groups were made using unpaired two-tailed Student's t-tests. For multiple group comparisons, one-way analysis of variance (ANOVA) was used. If the data are not normal or N is too small to determine normality (N<6), a non-parametric alternative is used. Detailed statistical information for each experiment can be found in Supplementary Table 1. P < 0.05 was considered statistically significant. *P < 0.05, **P < 0.01, ***P < 0.001, ****P < 0.0001.

## RESULTS

### Neurodegeneration, neuroinflammation and stress inhibit adult hippocampal neurogenesis

Previous research has documented that AHN is susceptible to various insults, including aging, endocrine disruptions, neuroinflammation and environmental toxins [5]. However, these factors may exert detrimental effects on AHN via distinct molecular and cellular mechanisms. In this study, we examined the AHN levels in four animal models of neurological diseases: the amyloid precursor protein (APP) and presenilin 1 (PS1) transgenic mice for Alzheimer’s disease, neuroinflammation induced by intracerebroventricular (i.c.v.) injection of lipopolysaccharide (LPS), learned helplessness (LH) and chronic restraint stress (CRS) to mimic stress and MDD (Supplementary Fig. 1).

APP/PS1 transgenic mouse model harbors familial mutations in both the APP (695swe) and PS1 (dE9) genes, exhibiting overproduction of amyloid-beta (Aβ), formation of plaque deposits and other pathological features characteristic of Alzheimer's disease. Aβ is pro-inflammatory and activates microglia. We assessed changes in AHN in APP/PS1 mice at 6 months of age by immunostaining of doublecortin (DCX), indicative of newly generated neurons in the hippocampus. The dentate gyrus (DG) of wild-type (WT) mice showed prominent DCX expression, while APP/PS1 mice exhibited a marked reduction in DCX^+^ signal (Fig. 1A). Quantitative analysis showed that, compared to the WT group, DG in APP/PS1 mice had significantly lower density of DCX^+^ cells (Fig. 1B), diminished dendritic branching (Fig. 1C) and a significant reduction in the dendritic length (Fig. 1D) of DCX^+^ neurons. These results indicated inhibited AHN under neurodegenerative conditions.

Next, we used i.c.v. injection of LPS to directly induce neuroinflammation and examine AHN. When compared to PBS injection, the LPS group showed reduced immunofluorescence intensity, indictive of decreased DCX expression (Fig. 1E). Statistical analysis found a significant decrease in DCX^+^ cell density (Fig. 1F), reduced dendritic branching (Fig. 1G) and a significant compromise in dendritic length (Fig. 1H) in LPS-treated mice.

Stress, unlike Aβ and LPS that directly activate immune cells and induce neuroinflammation, may affect AHN via endocrinological disruption. The LH model is an established paradigm in neuropsychological research for emulating a mental condition akin to acute stress. Mice were subjected to unavoidable and unpredictable electric shocks to the plantar surface to induce a state of LH. After shocks, avoidance was recorded if mice shuttled to the other compartment. Mice subjected to LH stress were categorized into LH group and non-learned helplessness (NLH) group, based on their behavioral responses during the LH test phase (Supplementary Fig. 2). Previous studies found that LH mice exhibit behavioral alterations similar to symptoms observed in human depression, including reduced voluntary activity, deficits in experiencing pleasure, sleep irregularities, and cognitive impairments [15].

**Figure 2.**
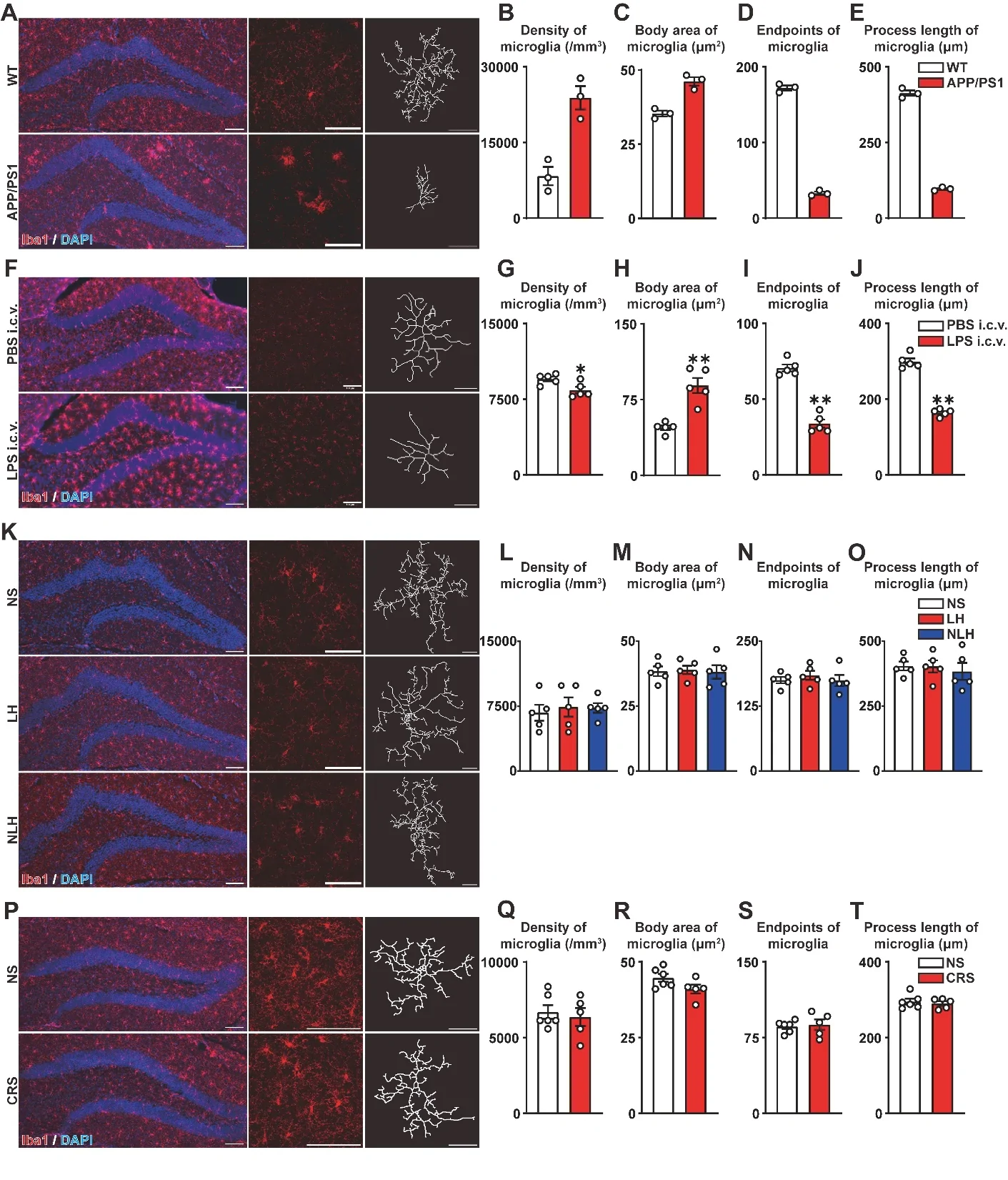
**Microglia activation is not involved in stress-induced inhibition of AHN**. (**A**) Hippocampal coronal sections of the brains of mice in the WT and APP/PS1 groups were stained with Iba1 antibody. Scale bar = 100 μm, Scale bar of morphological analysis = 20 μm. (B, C, D, E) Quantification of the density of Iba1^+^ microglia (B), average body area (C), number of endpoints (D), and average process length of microglia (E). n = 3 mice in each group. (**F**) Hippocampal coronal sections of the brains of mice in the PBS i.c.v. and LPS i.c.v. groups were stained with Iba1 antibody. Scale bar = 100 μm, Scale bar of morphological analysis = 20 μm. (G, H, I, J) Quantification of the density of Iba1^+^ microglia (G), average body area (H), number of endpoints (I), and average process length of microglia (J). n = 5 mice in each group. (**K**) Hippocampal coronal sections of the brains of mice in the NS, LH and NLH groups were stained with Iba1 antibody. Scale bar = 100 μm, Scale bar of morphological analysis = 20 μm. (L, M, N, O) Quantification of the density of Iba1^+^ microglia (L), average body area (M), number of endpoints (N), and average process length of microglia (O). n = 5-6 mice in each group. (**P**) Hippocampal coronal sections of the brains of mice in the NS and CRS groups were stained with Iba1 antibody. Scale bar = 100 μm, Scale bar of morphological analysis = 20 μm. (Q, R, S, T) Quantification of the density of Iba1^+^ microglia (Q), average body area (R), number of endpoints (S), and average process length of microglia (T). n = 5 mice in each group. For (B-E, G-J, L-O, Q-T), data are represented as mean ± SEM. Statistical analysis was performed using Kruskal-Wallis test (L-O), Mann Whitney test (B-E, G-J) and two-tailed unpaired t-tests (Q-T); *p < 0.05, **p < 0.01.

Next, we examined newly generated neurons in the DG of these mice by DCX immunostaining. Foot shocks did not change the density of DCX^+^ cells in either LH or NLH groups, compared to the NS group of mice (Fig. 1J). There were no differences between LH and NLH groups in cell morphology, but stress significantly reduced the branch number and dendritic length of DCX^+^ cells in both LH and NLH groups, when compared to the NS group (Fig. 1K, L). Interestingly, the impairment in DCX^+^ cell morphology appears to be independent of the behavioral phenotype in LH and NLH groups, indicating that the stress modeling itself is sufficient to inhibit neurogenesis. The behavioral manifestations of LH may correlate with other processes, such as plasticity of neural circuits.

Both LH and CRS models are commonly used in studies on stress. While the LH model primarily focuses on the psychological aspect of stress, the CRS model primarily focuses on the physiological effects of stress, particularly on the neuroendocrine and immune systems. In our experiments, mice subjected to 21 days of restraint stress, 2 hours per day, exhibited significantly reduced density of DCX^+^ cells, as well as branch number and dendritic lengths of DCX^+^ cells, compared to non-stressed (NS) group (Fig. 1M-P).

These results demonstrate that AHN can be affected by neurodegeneration, neuroinflammation, and both physiological and psychological stress.

**Figure 3.**
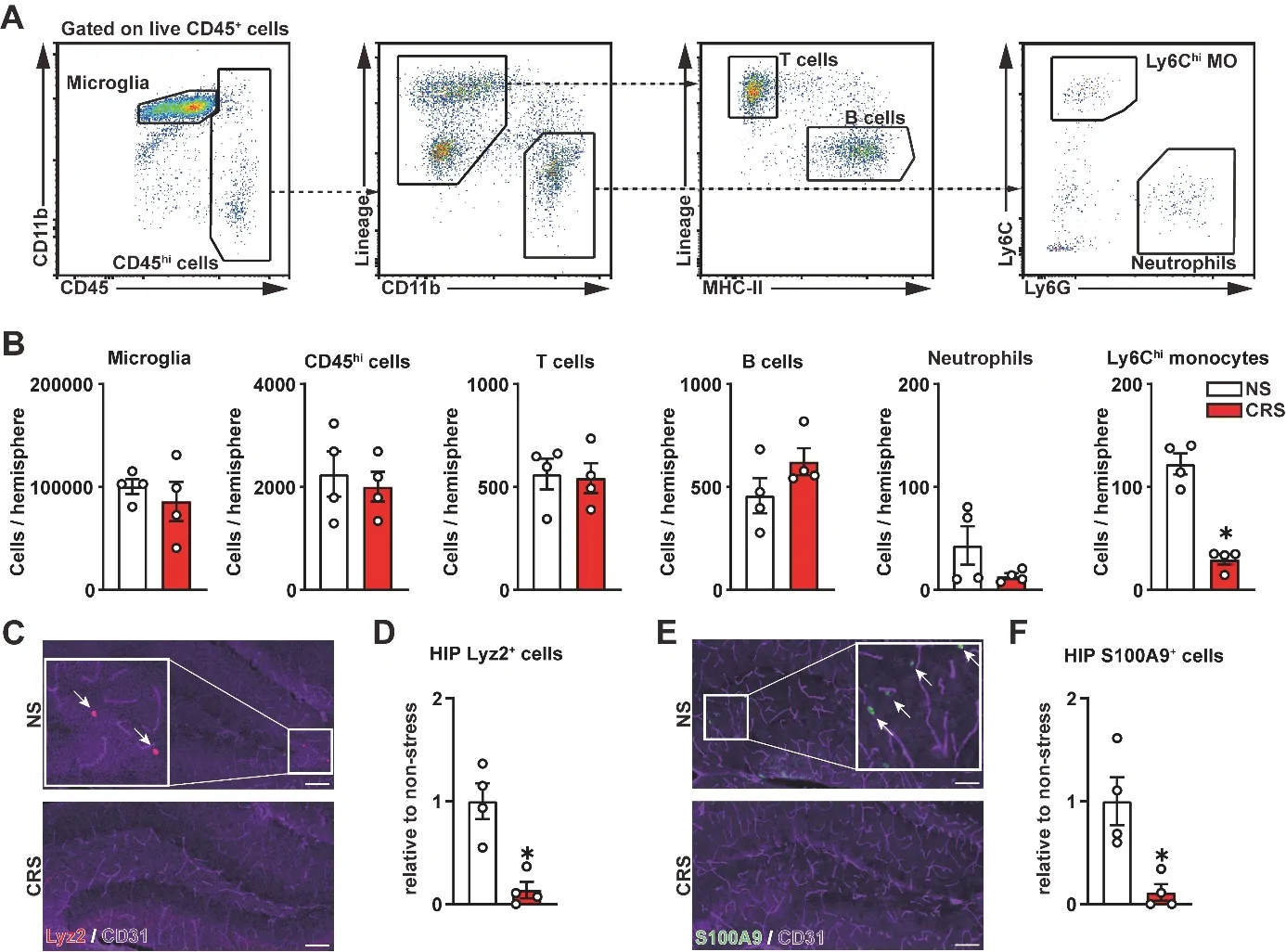
**CRS stress decreased resident Ly6C^hi^ monocytes in the hippocampal DG**. (**A**) Representative gating strategy of all major immune cell populations in the brain. Microglia are defined as CD45^int^ CD11b^+^, T cells are defined as CD45^hi^ CD11b^-^ Lineage^+^ MHC-II^-^, B cells are defined as CD45^hi^ CD11b^-^ Lineage^+^ MHC-II^+^, neutrophils are defined as CD45^hi^ CD11b^+^ Lineage^-^ Ly6G^+^ Ly6C^-^and Ly6C^hi^ monocytes are defined as CD45^hi^ CD11b^+^ Lineage^-^ Ly6G^-^ Ly6C^hi^. (**B**) Quantification of the absolute number of major immune cells in brain of mice in the NS and CRS groups. n = 4 mice in each group. (**C**) Representative hippocampal coronal sections of the brains of Lyz2-tdTomato mice in the NS and CRS groups were stained with CD31 antibody. Scale bar = 100μm. (**D**) Quantification of the relative number of Lyz2^+^ cells in hippocampus after CRS stress. n = 4 mice in each group. (**E**) Representative hippocampal coronal sections of the brains of mice in the NS and CRS groups were stained with S100A9 and CD31 antibody. Scale bar = 100μm. (**F**) Quantification of the relative number of S100A9^+^ cells in hippocampus after CRS stress. n = 4 mice in each group. For (B, D, F), data are represented as mean ± SEM. Statistical analysis was performed using Mann Whitney test; *p < 0.05.

### Microglia activation is not involved in stress-induced inhibition of AHN

Microglia play a multifaceted role in adult hippocampal neurogenesis (AHN), influencing various stages of neuron development and brain function [16]. They contribute to the maintenance of the neurogenic niche and support the proliferation, survival, and differentiation of neural progenitor cells into mature neurons [17]. When activated under pathological conditions, such as AD, neuroinflammation, and epilepsy, microglia become pro-inflammatory, phagocytic and release cytokines that impair neural stem cell functions and negatively affect the survival and integration of new neurons [18]. However, their role in AHN in response to various forms of stress remains unclear.

To investigate microglia activation in response to different insults and environmental stimuli that inhibit AHN, we immunostained Iba1, a marker for activated microglia, in the hippocampal DG region of four models shown in Figure 1. The cell density (Fig. 2B) and body area (Fig. 2C) of microglia in APP/PS1 transgenic mice significantly increased, whereas the number of endpoints (Fig. 2D) and process length (Fig. 2E) of microglia were significantly reduced, indicating a more activated and proliferative state associated with AD pathology. In LPS-treated mice, the cell density of microglia was slightly lower (Fig. 2G), but similar to microglia in APP/PS1 mice, the cell body area (Fig. 2H) significantly increased, while the endpoints (Fig. 2I) and process length (Fig. 2J) markedly decreased following intracerebroventricular (i.c.v.) LPS injection. These morphological alterations suggest that pro-inflammatory activation of microglia may contribute to the inhibition of AHN.

Next, we investigated whether microglial activation is implicated in stress-induced inhibition of AHN. Using Iba1 immunostaining, we assessed the cell density and morphology of microglia in the DG region of mice subjected to LH and CRS. As depicted in Figure 2K, after 2 days of foot shocks, neither LH nor NLH group exhibited any significant changes in microglial cell density or morphology. In the CRS model, mice were restrained for 21 days, 2 hours per day. Despite the prolonged stress paradigm, there were no significant differences in microglial density or morphological characteristics, such as cell body area, number of cell branches, and branch length in the DG (Fig. 2P-T). These findings demonstrate that the detrimental effects of stress on AHN are not associated with the activation state of microglia. Other mechanisms are likely underlying the inhibition of AHN by stress.

### CRS stress decreases resident Ly6C^hi^ monocytes in the hippocampal DG

It has been reported that peripheral pro-inflammatory immune cells may infiltrate the brain and reduce neurogenesis by suppressing neuronal stem cell proliferation, increasing the apoptosis of neuronal progenitor cells, thus decreasing the survival and integration of newly developing neurons into neuronal circuits [19]. Conversely, immune cells may enter the brain in healthy individuals and play essential roles in surveillance, protection, and response to injury, highlighting the dynamic interplay between the immune system and the central nervous system (CNS) for maintaining brain health and function [20]. To investigate the involvement of peripheral immune cells in stress-induced inhibition of AHN, we performed flow cytometry analysis on dissociated single cell suspensions of whole brains from NS and CRS mice (Fig. 3A).

The amount of microglia (CD45^int^ CD11b^+^), T cells (CD45^hi^ CD11b^-^ Lineage^+^ MHC-II^-^), B cells (CD45^hi^ CD11b^-^ Lineage^+^ MHC-II^+^), and neutrophils (CD45^hi^ CD11b^+^ Lineage^-^ Ly6G^+^ Ly6C^-^) were comparable between the NS and CRS brains (Fig. 3B). However, the number of brain-resident Ly6C^hi^ monocytes (CD45^hi^ CD11b^+^ Ly6G^-^ Ly6C^+^) was significantly reduced after 21 days of CRS treatment. To spatially map the resident Ly6C^hi^ monocytes within the brain parenchyma, particularly in the hippocampal DG region, we utilized confocal imaging on Lyz2-tdTomato mice. The Lyz2-tdTomato strain enables specific visualization of peripheral myeloid cell infiltration in the brain, as recruited myeloid cells exhibited high levels of tdTomato expression, while microglia in the CNS do not [21]. In Lyz2-tdTomato mice, as confirmed by flow cytometry, tdTomato was highly expressed in Ly6C^hi^ monocytes and neutrophils, but not in T cells or B cells (Supplementary Fig. 3). Next, we used CD31 immunofluorescence staining as the marker for endothelial cells in blood vessels. Infiltrated tdTomato^+^ cells were observed in the hippocampus of the NS group. Compared to the NS group, the CRS group exhibited a significantly decreased number of tdTomato^+^ cells in the hippocampal DG region (Fig. 3C, D). Since Lyz2 promoter is active in both monocytes and neutrophils, we further confirmed the identity of these tdTomato^+^ cells as monocytes, by immunostaining S100A9, a marker for infiltrating monocytes in various conditions and tissues [22]. Consistent with the Lyz2 results, the CRS group showed a significantly decreased number of S100A9^+^ cells (Fig. 3E, F). In summary, CRS stress specifically reduced resident monocytes in the brain.

**Figure 4.**
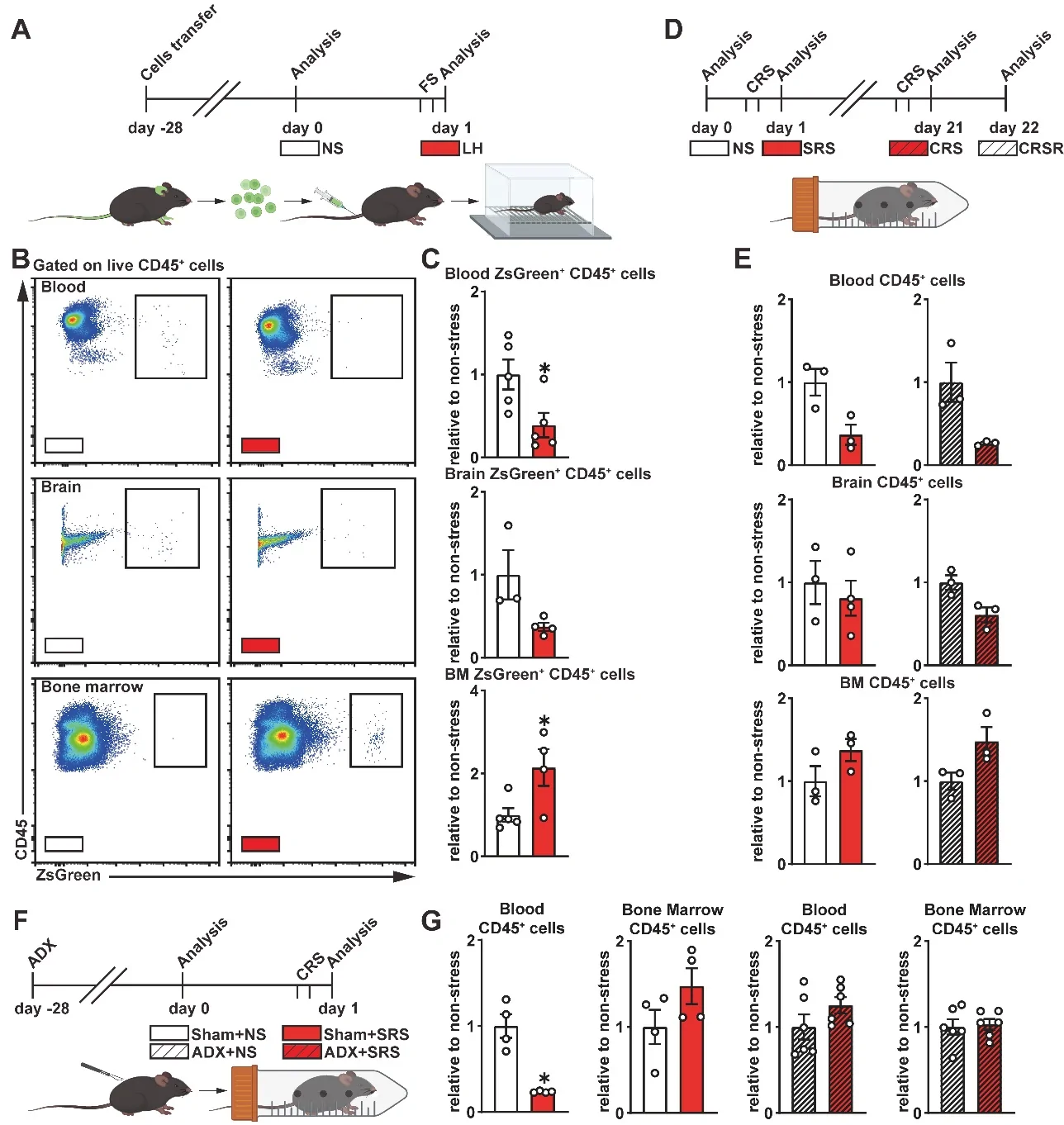
**Stress drives the homing of peripheral immune cells to the bone marrow**. (**A**) Schematic overview of LH stress followed by cells transfer. (**B**) Representative flow-cytometry plots of transferred cells in blood, brain, and bone marrow of recipient mice after LH stress. Transferred cells are defined as CD45^+^ ZsGreen^+^. (**C**) Quantification of the relative number of transferred cells in blood, brain, and bone marrow of recipient mice after LH stress. n = 3-5 mice in each group. (**D**) Schematic overview of SRS and CRS stress. (**E**) Quantification of the relative number of CD45^+^cells in blood, brain and bone marrow of mice in the NS, SRS, CRSR and CRS groups. n = 3 mice in each group. (**F**) Schematic overview of SRS followed by bilateral adrenalectomy (ADX). (**G**) Mice underwent ADX or sham surgery and were allowed to recover for 4 weeks prior to submission to SRS Quantification of the relative number of CD45^+^cells in blood, brain, and bone marrow of mice in the Sham + NS, Sham + SRS, ADX+NS and ADX+SRS groups. n = 4-6 mice in each group. For (C, E, G), data are represented as mean ± SEM. Statistical analysis was performed using Mann Whitney test (C, E, G - Sham+NS (4) & Sham+SRS (4)) and two-tailed unpaired t-tests (G - ADX+NS (6) & ADX+SRS (6)); *p < 0.05.

**Figure 5.**
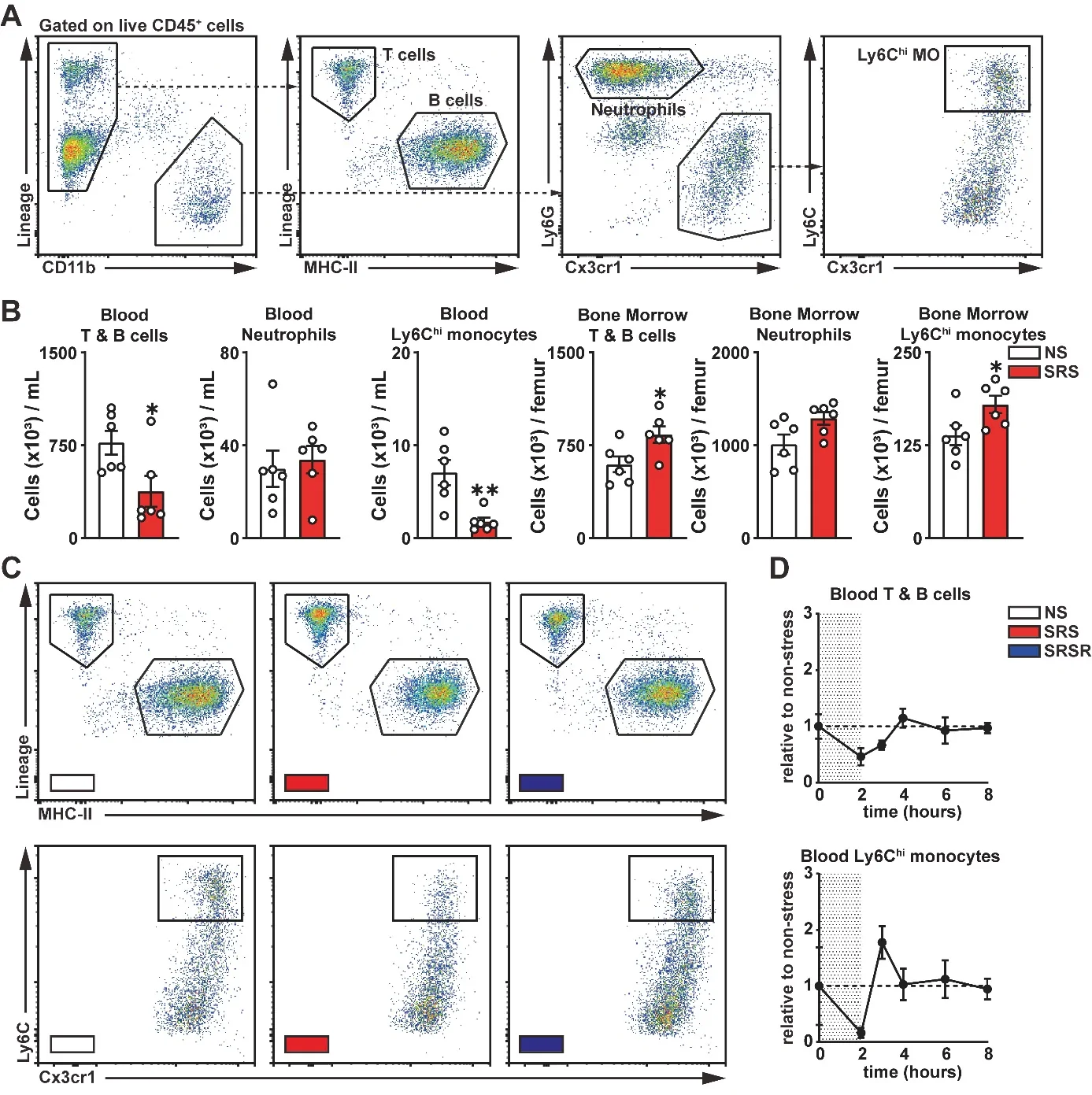
**Stress drastically diminished Ly6C^hi^ monocytes in peripheral blood**. (**A**) Representative gating strategy of all major immune cell populations in the blood. T cells are defined as CD45^+^ CD11b^-^ Lineage^+^, B cells are defined as CD45^+^ CD11b^-^ Lineage^+^ MHC-II^+^, neutrophils are defined as CD45^+^ CD11b^+^ Lineage^-^ Cx3cr1^-^ Ly6G^+^ and Ly6C^hi^ monocytes are defined as CD45^+^ CD11b^+^ Lineage^-^ Cx3cr1^+^ Ly6C^hi^. (**B**) Quantification of the absolute number of major immune cells in blood and bone marrow of mice in the NS and SRS groups. n = 6 mice in each group. (**C**) Representative flow-cytometry plots of T&B cells and Ly6C^hi^ monocytes in the blood of mice in the NS, SRS and SRSR groups. (**D**) T&B cells and Ly6C^hi^ monocytes numbers in blood measured after the indicated time of recovery from single restraint stress episode, expressed as relative of mice in the NS group. n = 3 mice in each group. For (B, D), data are represented as mean ± SEM. Statistical analysis was performed using two-tailed unpaired t-tests (B) and Kruskal-Wallis test (D); *p < 0.05, **p < 0.01.

### Stress induces glucocorticoid-dependent homing of brain immune cells to the bone marrow

Immune cells are known to redistribute in response to various stimuli. Following chemotactic gradients, immune cells migrate to specific tissues or organs. It is well established that stress effectively drives immune cells from the blood stream to actively cross the blood-bone marrow endothelial barrier and lodge in the bone marrow compartment, known as the “homing” [23]. However, whether brain-resident immune cells also exhibit homing behavior to bone marrow remains unclear.

To investigate whether this mechanism might account for the reduction of brain monocytes after CRS shown in Figure 3, we adoptively transferred immune cells of ZsGreen^+^ mice to WT mice. Since ZsGreen^+^ immune cells were transferred intravenously, their appearance in the bone marrow necessarily required migration from blood. Previous study has demonstrated that adoptively transferred allogenic immune cells became resident in the brain of recipient mice [24]. Twenty-eight days after the cell transfer, we applied 2 days of inescapable foot shock, the LH procedure, and measured ZsGreen+ immune cell subpopulations in the blood, the brain, and the bone marrow (Fig. 4A, B). The results show that the CD45^+^ immune cells were significantly reduced in both the brain and blood, accompanied by a more than two-fold increase in the bone marrow (Fig. 4C), demonstrating that immune cells in the brain redistribute similar to the cells in blood, suggesting bone marrow homing.

**Figure 6.**
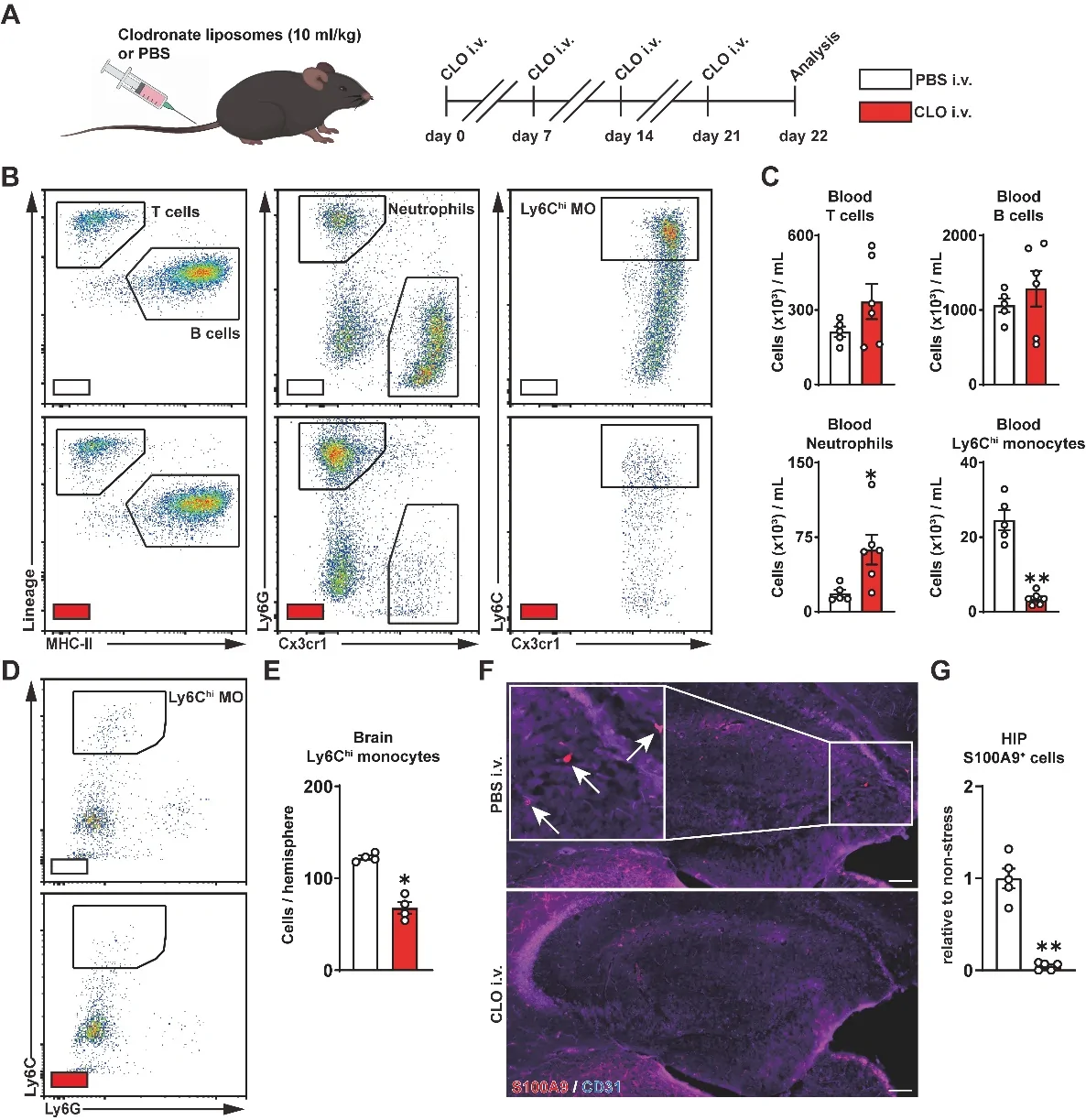
**Depletion of circulating monocytes decreases numbers of resident Ly6C^hi^ monocytes in the hippocampal DG**. (**A**) Schematic overview of depletion of circulating monocytes. (**B**) Representative gating strategy of all major immune cell populations in the blood of mice in the PBS i.v. and CLO i.v. groups. T cells are defined as CD45^+^ CD11b^-^ Lineage^+^, B cells are defined as CD45^+^ CD11b^-^ Lineage^+^ MHC-II^+^, neutrophils are defined as CD45^+^ CD11b^+^ Lineage^-^ Cx3cr1^-^ Ly6G^+^ and Ly6C^hi^ monocytes are defined as CD45^+^ CD11b^+^ Lineage^-^ Cx3cr1^+^ Ly6C^hi^. (**C**) Quantification of the absolute number of major immune cells in the blood of mice in the PBS i.v. and CLO i.v. groups. n = 5-6 mice in each group. (**D**) Representative flow-cytometry plots of Ly6C^hi^ monocytes in the brain of mice in the PBS i.v. and CLO i.v. groups. (**E**) Quantification of the absolute number of Ly6C^hi^ monocytes in the brain of mice in the PBS i.v. and CLO i.v. groups. n = 4 mice in each group. (**F**) Hippocampal coronal sections of the brains of mice in the PBS i.v. and CLO i.v. groups were stained with S100A9 and CD31 antibody. Scale bar = 100μm. (**G**) Quantification of the relative number of S100A9^+^ cells in hippocampus after depletion of circulating monocytes. n = 5 mice in each group. For (C, E, G), data are represented as mean ± SEM. Statistical analysis was performed using two-tailed unpaired t-tests (C) and Mann Whitney test (E, G); ^*^p < 0.05, ^**^p < 0.01

**Figure 7.**
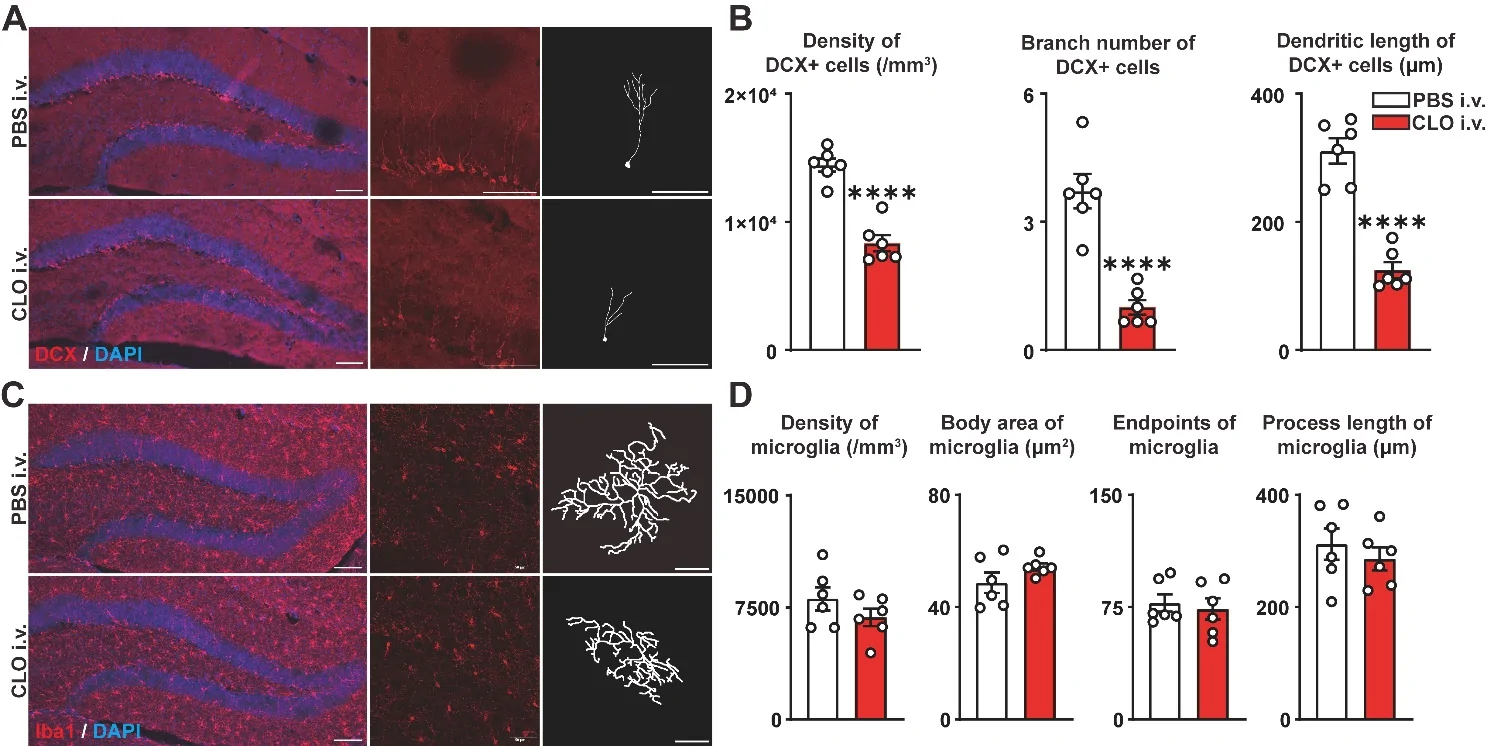
**Depletion of circulating monocytes reproduced the inhibitory effects of AHN by stress**. (**A**) Hippocampal coronal sections of the brains of mice in the PBS i.v. and CLO i.v. groups were stained with DCX antibody. Scale bar = 100μm, Scale bar of morphological analysis = 100 μm. (**B**) Quantification of the density of DCX^+^ newborn immature neuron, average dendritic branch number and average dendritic branch length. n = 6 mice in each group. (**C**) Hippocampal coronal sections of the brains of mice in the PBS i.v. and CLO i.v. groups were stained with Iba1 antibody. Scale bar = 100μm, Scale bar of morphological analysis = 20 μm. (**D**) Quantification of the density of Iba1^+^ microglia, average body area, number of endpoints and average process length of microglia. n = 6 mice in each group. For (B, D), data are represented as mean ± SEM. Statistical analysis was performed using two-tailed unpaired t-tests; ****p < 0.0001.

Furthermore, we investigated whether chronic stress, such as 21 days of CRS, induces a similar pattern of homing. To address this question, we compared mice subjected to single restraint stress (SRS) with non-stressed (NS) mice, and mice subjected to CRS with those subjected to CRS followed by one day of recovery (CRSR) (Fig. 4D). As shown in Fig. 4C, single restraint stress led to a decrease in ZsGreen^+^ cells in the blood. After 21 days of daily restraint stress treatment, ZsGreen^+^ cells were significantly reduced in both the brain and blood, while increasing in the bone marrow (Fig. 4E). This result aligns with our findings from the LH experiment, suggesting that both acute (LH) and chronic (CRS) stress induce homing of immune cells from both the blood and the brain to the bone marrow.

The homing of immune cells is a tightly regulated process governed by glucocorticoids released by the adrenal gland in response to stress, e.g. fasting [23]. To confirm the role of adrenal gland in our stress models, we performed bilateral adrenalectomy (ADX) in mice prior to SRS and found that homing of CD45^+^ immune cells was prohibited in the absence of adrenal glands (Fig. 4F, G). This result verifies that the observed immune cell homing is glucocorticoid-dependent and follows conventional homing behavior.

### Stress causes a drastic reduction of Ly6C^hi^ monocytes in the peripheral blood

To further investigate the effects of stress on immune cells homing at the subpopulation level, we applied SRS to mice and measured the numbers of T cells, B cells, neutrophils, and monocytes in blood and bone marrow by flow cytometry. The results showed a significant reduction in the number of T cells, B cells and Ly6C^hi^ monocytes in the peripheral blood, but significantly increased in the bone marrow in the SRS group (Fig. 5A, B). In line with the finding that monocytes, but not neutrophils, were diminished from hippocampal DG region after stress (Fig. 3D, F), the numbers of neutrophils in blood and bone marrow remained unchanged (Fig. 5A, B). To assess the kinetics of these changes, we conducted time-course experiments. We observed a significant reduction in the number of T cells, B cells and Ly6C^hi^ monocytes in peripheral blood in the SRS group compared to the NS group, with all cell populations returning to baseline levels after 6 hours of recovery. However, while T and B cell numbers recovered completely, Ly6C^hi^ monocytes were almost completely depleted in response to stress, suggesting a more persistent homing behavior that may contribute to the decrease in resident monocytes within the brain.

To further explore the temporal dynamics of stress-induced changes in monocyte distribution and neurogenesis, we conducted a time-course experiment to elucidate the onset, progression, and reversibility of these effects. Brains from mice subjected to various durations of CRS were collected. We found that while microglia numbers remained relatively stable, non-microglial immune cells in the brain decreased during the first 14 days of CRS, returning to baseline levels before stress withdrawal (Supplementary Fig. 4A-C). T cells and B cells, which constitute most non-microglial immune cells, also returned to baseline levels before stress withdrawal (Supplementary Fig. 4D-F). In contrast, myeloid cells exhibited a distinct response, with their numbers decreasing to a low point during the stress period and recovering to baseline levels 14 days after stress withdrawal. Among these myeloid cells, Ly6C^hi^ monocytes showed a significant decrease (Supplementary Fig. 4G-I), which correlated with the impairment and subsequent recovery of AHN (Supplementary Fig. 4J-M). These results suggest a strong correlation between stress-induced impairment of AHN and the reduction of Ly6C^hi^ monocytes in the brain, with both effects exhibiting a reversible nature.

### Depletion of circulating monocytes eliminates Ly6C^hi^ monocytes in the hippocampal DG and inhibits AHN

To examine whether the stress-induced elimination of Ly6C^hi^ monocytes in the peripheral blood affects monocyte infiltration into the hippocampal DG region, we administered clodronate liposomes (CLO) intravenously (i.v.) to deplete peripheral monocytes. Mice received CLO injections every 7 days for a total of 21 days (Fig. 6A). The depletion efficiency of Ly6C^hi^ monocytes was measured using flow cytometry. Results showed that the absolute number of Ly6C^hi^ monocytes in peripheral blood was reduced by about 90% in the CLO i.v. group compared with the PBS group (Fig. 6B-C), while the numbers of T cells and B cells remained unchanged (Fig. 6A-B). Consistent with previous studies, neutrophil numbers increased [25]. Importantly, monocyte depletion significantly reduced the absolute number of Ly6c^hi^ monocytes in the hippocampus, as demonstrated by both flow cytometry (Fig. 6D, E) and immunofluorescence staining (Fig. 6F, G).

The results presented above demonstrate that CLO treatment reproduced the elimination effects of stress on circulating Ly6C^hi^ monocytes in the blood and on infiltrated monocytes in the hippocampus. Utilizing this approach, we assessed the effects of monocyte depletion on AHN. As shown in Fig. 7, when compared with the PBS group, the density of DCX^+^ cells in the hippocampal DG region of CLO-treated mice significantly decreased. These DCX^+^ cells also exhibited significantly impaired dendritic morphology in branch number and dendritic length (Fig. 7 A, B). Meanwhile, CLO-induced depletion of monocytes did not affect microglia in the hippocampal DG region. The CLO group did not show abnormal microglial proliferation, activation, or morphology, as assessed by endpoints and process length (Fig. 7C, D).

### CRS alters the transcriptional signature of blood Ly6C^hi^ monocytes.

**Figure 8.**
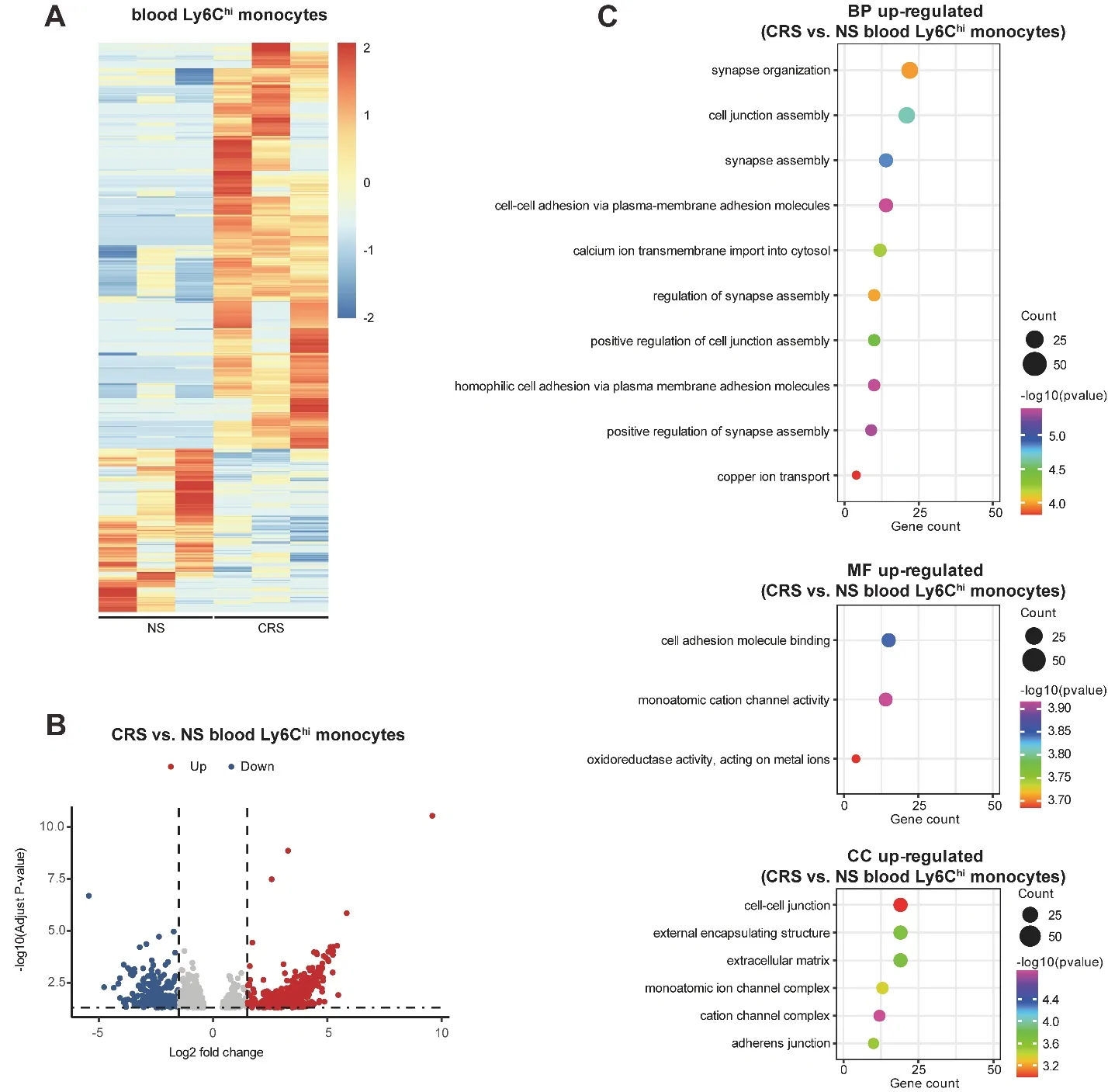
**CRS changed the transcriptional signature in Ly6C^hi^ monocytes**. (**A**) Heatmap of differentially expressed genes of sorted blood Ly6C^hi^ monocytes after CRS stress. (n = 3 samples per group, 5 mice pooled per sample). (**B**) Volcano plot indicating differentially regulated genes (FC > |1.5|, p < 0.05 and was detected in at least two samples) of sorted blood Ly6C^hi^ monocytes after CRS stress. (n = 3 per group, 5 mice pooled per sample). (**C**) Top gene ontology (GO) terms from significantly upregulated genes in Ly6C^hi^ monocytes of CRS versus NS mice.

To investigate transcriptional changes in Ly6C^hi^ monocytes induced by CRS, we performed fluorescence-activated cell sorting (FACS) to isolate Ly6C^hi^ monocytes from NS and CRS mice, followed by bulk RNA sequencing. The results revealed significant transcriptional alterations in this monocyte subset, with a total of 759 differentially expressed genes (DEGs) identified between CRS and NS mice (P < 0.05 and fold change (FC) > |1.5|). Among these DEGs, 541 genes were upregulated, and 218 were downregulated (Fig. 8A, B). Gene ontology (GO) enrichment analysis, focusing on biological processes (BP), cellular components (CC), and molecular functions (MF), revealed that the upregulated genes in CRS mice were associated with biological processes such as synapse organization, cell junction assembly, synapse assembly, and cell-cell adhesion mediated by plasma membrane adhesion molecules. In terms of cellular components, these genes were linked to cell-cell junctions, external encapsulating structures, and extracellular matrix. Additionally, they were involved in molecular functions such as cell adhesion molecule binding (Fig. 8C). Collectively, the upregulated DEGs in monocytes from the CRS group suggest supportive effects on neuronal functions. By enhancing synaptic organization, cell adhesion, and the extracellular environment, these upregulated genes may support the survival and functional integration of new neurons within the hippocampus.

## DISCUSSION

In this study, we demonstrated that neurodegenerative and neuroinflammatory conditions, as well as stress induced by both LH and CRS, impaired AHN, as evidenced by changes in DCX^+^ cell density and cell morphological characteristics (Fig. 1). However, the activation of microglia, the primary source of neuroimmunological modulation, varied significantly among these conditions. While AD transgenic mice and LPS-induced acute neuroinflammation significantly activated microglia, stress induced by LH and CRS did not (Fig. 2). By measuring the proportions of brain-resident immune cells, circulating immune cells, and bone marrow resident cells, we identified that stress markedly and specifically reduced Ly6C^hi^ monocytes in both the hippocampal DG region and the blood (Fig. 3-5). In response to restraint stress, these Ly6C^hi^ monocytes migrated to the bone marrow in a glucocorticoid-dependent manner, as evidenced by the adrenalectomy experiment (Fig. 4). Depletion of monocytes by peripheral CLO treatment reduced resident monocytes in the hippocampus and reproduced the stress-induced inhibition of AHN (Fig. 6-7). Bulk RNA sequencing revealed significant transcriptional changes in blood Ly6C^hi^ monocytes induced by CRS. GO enrichment analysis indicated upregulated genes were associated with neural protection (Fig. 8). Collectively, these results indicate a novel role for brain-resident Ly6C^hi^ monocytes in maintaining AHN and brain functions. Stress-induced bone marrow homing of circulation and brain-resident monocytes may impair AHN and may contribute to aging-related disorders.

To date, multiple mechanisms are known to affect AHN. At the cellular level, it is well established that microglia and astrocytes play pivotal roles in regulating neuronal integrity and neurogenesis processes in response to injuries, inflammatory responses, and neurodegenerative lesions [26]. *In vitro* studies have shown that conditioned medium from LPS-activated microglia cultures can impair neuronal differentiation [27], suggesting that inflammatory factors released by microglia directly influence the neuronal differentiation. Furthermore, *in vivo* experiments show that reducing microglia activation, through the administration of LPS followed by anti-inflammatory or neuroprotective drugs, positively correlates with restored hippocampal neurogenesis [28]. At the molecular level, pro-inflammatory cytokines are increasingly recognized for their roles in various stages of neurogenesis, with the hippocampus being particularly susceptible [29]. NSCs in the DG region express receptors for cytokines and chemokines, making them primary targets for pro-inflammatory signaling pathways [30]. Transgenic mice overexpressing IL-6 exhibit a significant decrease in the proliferation, differentiation, and survival of hippocampal neural precursor cells. Consistently, adult rat hippocampal precursor cells exposed to IL-6 show a marked reduction in proliferation [27]. Similarly, systemic administration of tumor necrosis factor-alpha (TNF-α) has been shown to impair cell proliferation in the adult hippocampus. *In vitro* neurogenesis assays indicate that TNF-α treatment not only decreases cell proliferation but also leads to a reduction in DCX^+^ cells, indicating impaired neuronal differentiation and neurogenesis [31].

Various forms of stress can have profound psychological and physiological effects, significantly impacting adult neurogenesis, particularly the proliferation of newborn neurons in the hippocampus [32-35]. Chronic stress has been implicated as a contributing factor to the pathogenesis of neurodegenerative diseases such as Alzheimer’s and Parkinson’s disease. It may also increase vulnerability to neuropsychiatric disorders, as reduced neurogenesis has been associated with conditions like depression and anxiety. In this study, the observed reduction in Ly6C^hi^ monocytes due to stress-induced bone marrow homing could be linked to impaired neurogenesis, which may be crucial for cognitive function. Stress activates the HPA axis, triggering the release of glucocorticoids from the adrenal cortex. Glucocorticoids cross the blood-brain barrier and bind to mineralocorticoid receptors (MRs) and glucocorticoid receptors (GRs) in the brain. Most type 1 neural stem cells (NSCs) and type 2a amplifying neural progenitor cells in the hippocampus express GRs, which exhibit varying expression levels across different stages of neuronal development. GRs function as transcriptional activators or repressors, thereby modulating gene transcription in newborn neurons [36, 37]. Consequently, glucocorticoids differentially regulate newborn neurons at distinct stages of AHN during stress. In addition to glucocorticoids, pro-inflammatory cytokines and neurotrophic factors, such as IL-1β and VEGF, also affect AHN [38-40].

From the perspective of bone marrow homing of peripheral immune cells, both MDD in humans and chronic stress in rodents have been associated with monocytosis, neutrophilia, and lymphopenia, which may result from altered proliferation of hematopoietic stem cells or mobilization of leukocytes in the bone marrow. However, it is important to note that the LH model and the CRS model used in this study differ significantly in their mechanisms and outcomes, particularly concerning neurogenesis. The primary distinction between these two models lies in the duration and nature of the stressors. The CRS model typically results in a greater loss of newborn neurons compared to the LH model. This difference is likely due to the chronic nature of stress in the CRS model, which leads to sustained activation of stress pathways and neurotoxic effects. In contrast, while the LH model involves severe acute stress, it may not engage the same prolonged neurobiological processes. Mice exposed to chronic variable stress or chronic social defeat stress exhibit myeloid progenitor cell expansion and lymphoid progenitor cell downregulation [41, 42]. The bone marrow receives dense sympathetic innervation, which contributes to the mobilization of leukocytes into the circulation under stress or homeostatic conditions [43]. Mechanistically, these sympathetic fibers release norepinephrine signals within the bone marrow that inhibit the expression of CXCL12, a chemokine that normally suppresses hematopoiesis and retains neutrophils and monocytes within the bone marrow [44]. Repeated social defeat stress has also been reported to decrease CXCL12 expression in the bone marrow, thereby increasing the release of monocytes, a phenomenon that is blocked by adrenalectomy or treatment with the corticosterone synthesis inhibitor metyrapone [45].

Several previous studies have reported an increase in peripheral monocyte/macrophage infiltration under stress conditions [46, 47], but our study reports a decrease. Prior research was limited to observing changes in the proportion of monocytes/macrophages among immune cells in the brain due to stress. We believe these proportional increases were attributable to a decrease in the absolute number of lymphocytes. In contrast, our study measured the absolute numbers of all types of immune cells in the brain under stress. These findings were validated using an immune cell transfer model and transgenic reporter mice (Fig. 4).

Regarding the supportive role of monocytes in neurogenesis, our study aligns with the findings of Möhle et al. They investigated the potential synergistic effects of the gut microbiota and physical exercise on AHN and proposed that these effects might be mediated by Ly6C^hi^ monocytes [48]. By co-culturing primary hippocampal NPCs with bone marrow-derived Ly6C^hi^ monocytes, they found a significant increase of neurosphere number, demonstrating that blood-derived monocytes are involved in maintaining neurogenesis in the uninjured CNS.

Lastly, we have demonstrated a novel and essential role for Ly6C^hi^ monocytes in maintaining AHN, which can be disrupted by stress-induced bone marrow homing. Future studies may address important questions raised by this research. For example, which chemokines mediate the stress-induced homing of brain-resident monocytes, and how do monocytes influence neurogenic niches? How are other brain-resident cells, such as microglia, neurons, and astrocytes, impacted by Ly6C^hi^ monocyte infiltration, and how do they participate in maintaining AHN?

## Supplementary material

The Supplementary data can be found online at: www.aginganddisease.org/EN/10.14336/AD.2023.0835.
