# Supplementary material for: Brain Resident Ly6Chi Monocytes Are Necessary for Maintaining Adult Hippocampal Neurogenesis
Source: Aging Dis. 2024 Nov 18;16(5):3069–88. doi: 10.14336/AD.2024.0835 (PMC12339147; doi:10.14336/AD.2024.0835)
Supplement: Supplementary file 1 — The Supplementary data can be found online at: www.aginganddisease.org/EN/10.14336/AD.2023.0835. [file AD-16-5-3069-s.pdf]

SUPPLEMENTARY DATA

Brain Resident Ly6C^{hi} Monocytes Are Necessary for Maintaining Adult Hippocampal Neurogenesis

Yiran Huang, Nan Gao, Boren Liu, Weili Luo, Jianfei Chen, Yan Chen, Yong Bi, Zikai Zhou

© 2024. Huang Y et al. Published online at http://www.aginganddisease.org/EN/10.14336/AD.2024.0835

SUPPLEMENTARY DATA

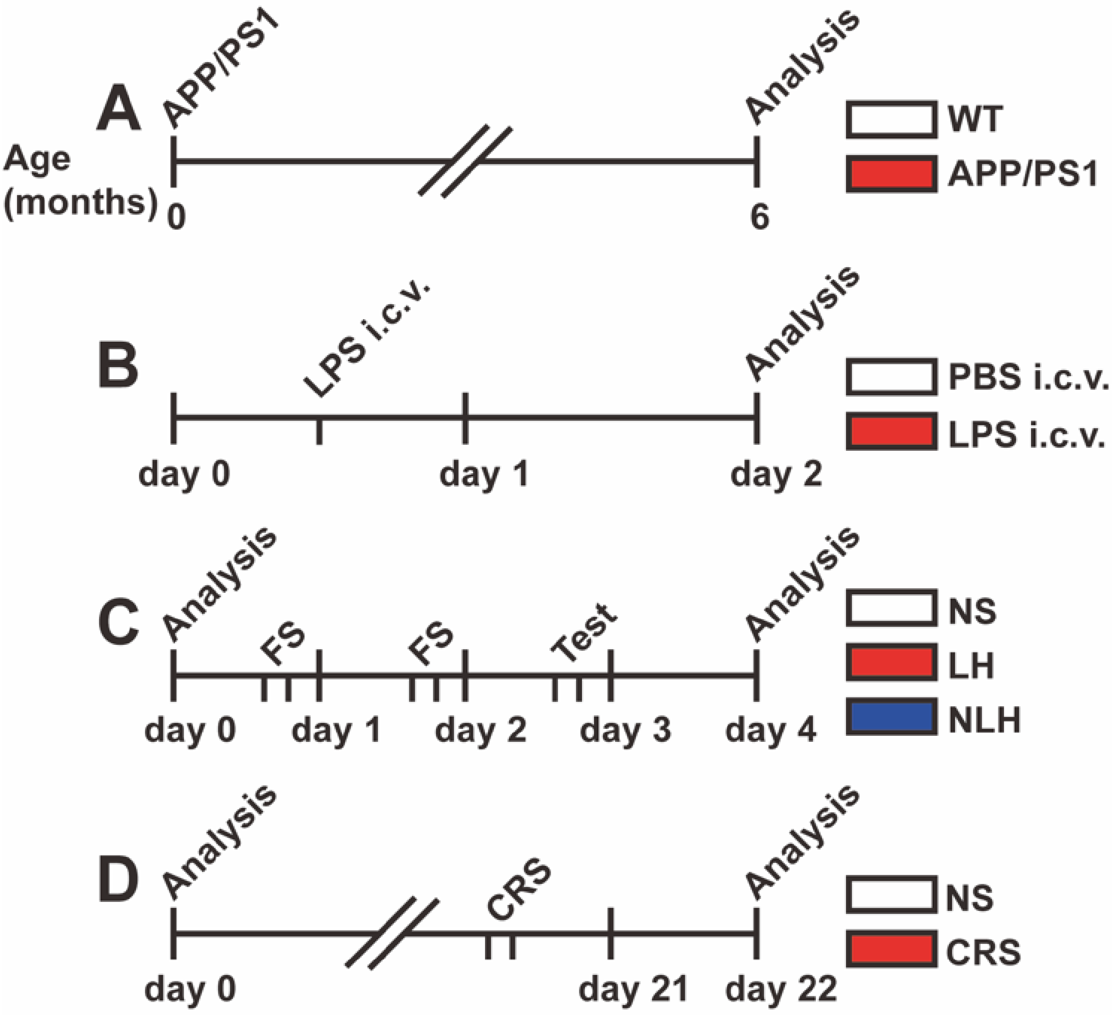

Supplementary Figure 1. Schematic overview of animal models used in Fig 1, related to Figure 1.

© 2024. Huang Y et al. Published online at http://www.aginganddisease.org/EN/10.14336/AD.2024.0835

SUPPLEMENTARY DATA

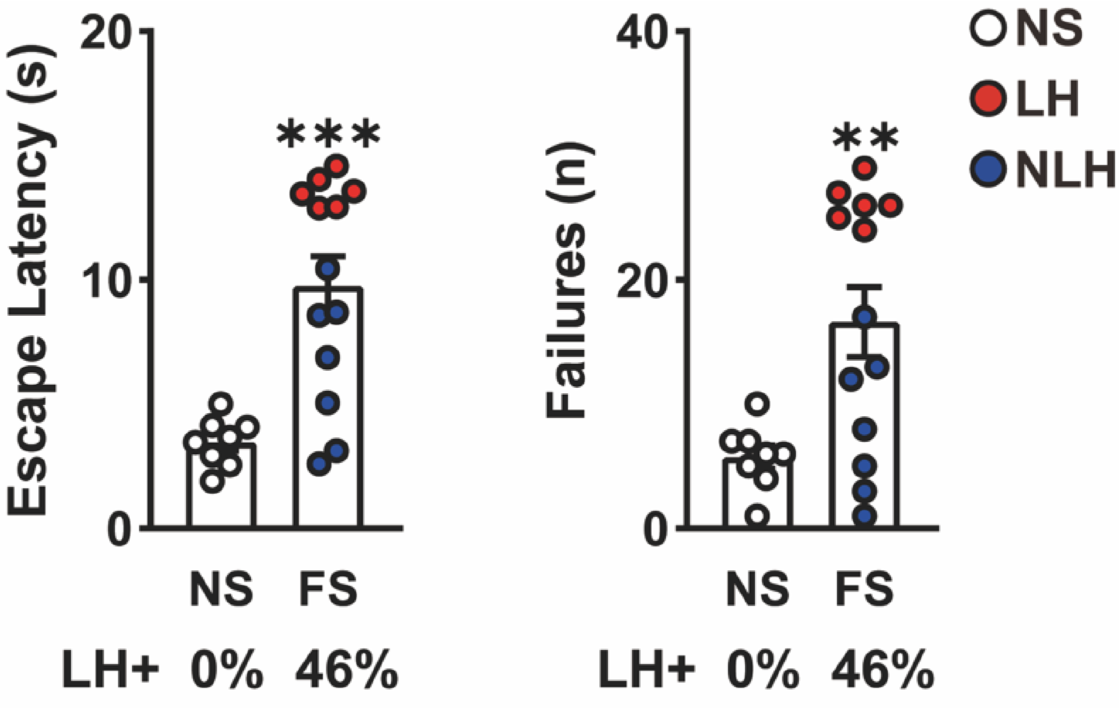

Supplementary Figure 2. Behavioral analysis of learned helplessness, related to Figure 1.

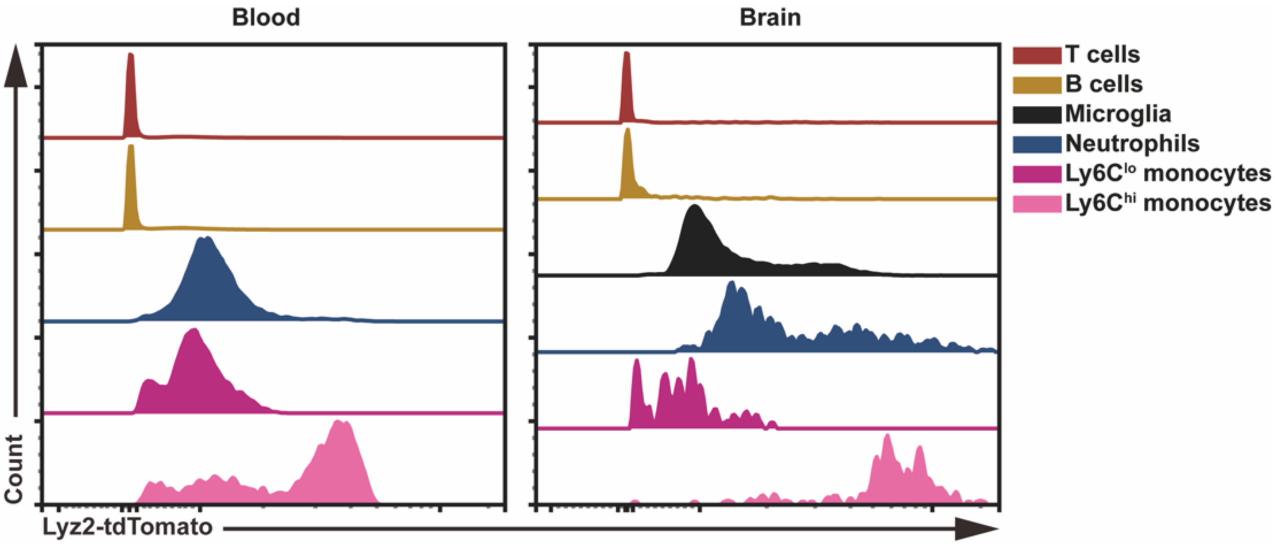

Supplementary Figure 3. tdTomato expression of blood and brain immune cells in Lyz2-tdTomato mice, related to Figure 3.

© 2024. Huang Y et al. Published online at http://www.aginganddisease.org/EN/10.14336/AD.2024.0835

SUPPLEMENTARY DATA

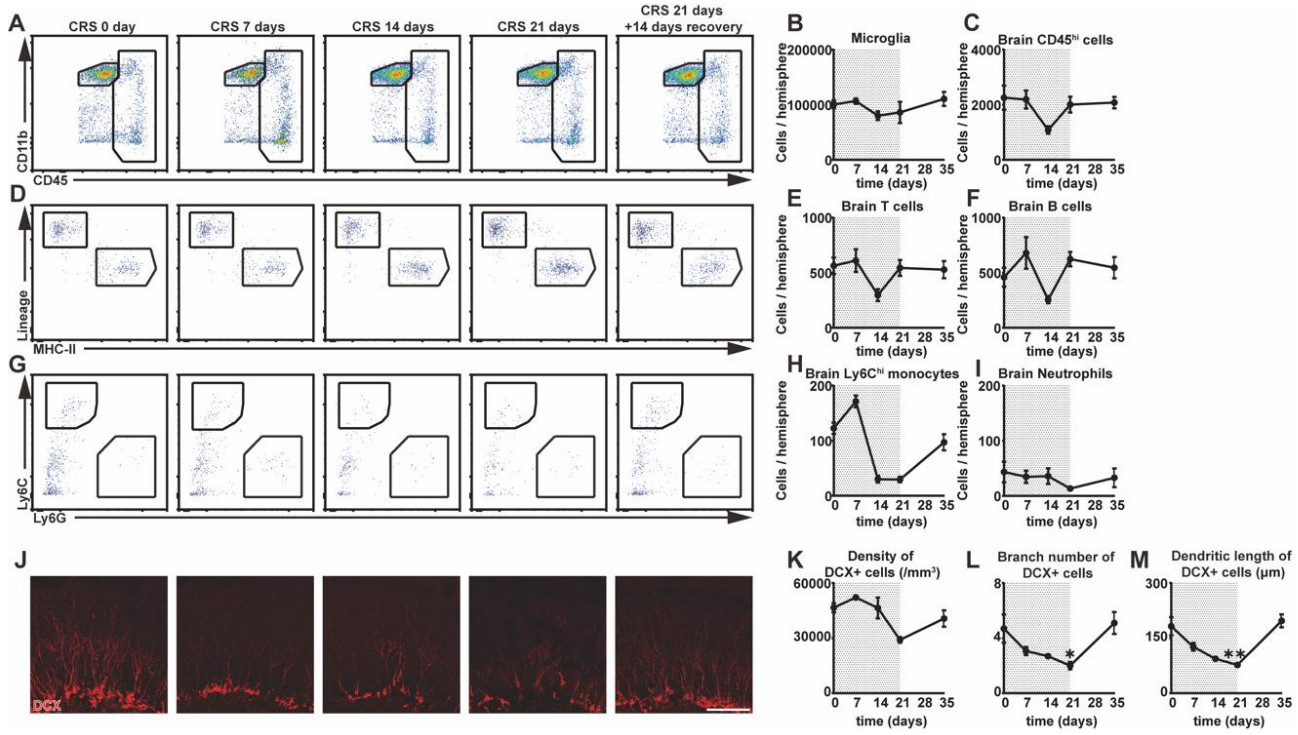

Supplementary Figure 4. The temporal dynamics of CRS stress effects brain immune cells distribution and neurogenesis. (A, D, G) Representative gating strategy of all major immune cell populations in the brain at each time point in CRS stress. Microglia are defined as $CD45^{int}$ $CD11b^{+}$, Non-microglial immune cells are defined as $CD45^{hi}$, T cells are defined as $CD45^{hi}$ $CD11b^{-}$ $Lineage^{+}$ MHC-II^{-}, B cells are defined as $CD45^{hi}$ $CD11b^{-}$ $Lineage^{+}$ MHC-II^{+}, neutrophils are defined as $CD45^{hi}$ $CD11b^{+}$ $Lineage^{-}$ $Ly6G^{+}$ $Ly6C^{-}$and $Ly6C^{hi}$ monocytes are defined as $CD45^{hi}$ $CD11b^{+}$ $Lineage^{-}$ $Ly6G^{-}$ $Ly6C^{hi}$. (B, C, E, F, H, I) Quantification of the absolute number of major immune cells in brain of mice at each time point in CRS stress. n = 4 mice at each time point. (J) Representative hippocampal coronal sections of the brains of mice at each time point in CRS stress were stained with DCX antibody. Scale bar = 100 μm. (K, L, M) Quantification of the density of DCX^{+} newborn immature neuron (K), average dendritic branch number (L), and average dendritic branch length (M). n = 4 mice at each time point. For (B, C, E, F, H, I, K, L, M), data are represented as mean ± SEM. Statistical analysis was performed using Kruskal-Wallis test. $^{*}p < 0.05$, $^{**}p < 0.01$.

© 2024. Huang Y et al. Published online at http://www.aginganddisease.org/EN/10.14336/AD.2024.0835
